# Dispersive Raman spectroscopy allows the identification and quantification of melanin types

**DOI:** 10.1002/ece3.1453

**Published:** 2015-03-04

**Authors:** Ismael Galván, Alberto Jorge

**Affiliations:** 1 Departamento de Ecología Evolutiva, Estación Biológica de Doñana - CSICc/Américo Vespucio s/n, 41092, Sevilla, Spain; 2 Museo Nacional de Ciencias Naturales - CSICc/José Gutiérrez Abascal 2, 28006, Madrid, Spain

**Keywords:** Eumelanin, feathers, pheomelanin, Raman spectroscopy

## Abstract

Melanins are the most prevalent pigments in animals and are involved in visual communication by producing colored traits that often evolve as intraspecific signals of quality. Identifying and quantifying melanins are therefore essential to understand the function and evolution of melanin-based signals. However, the analysis of melanins is difficult due to their insolubility and the lack of simple methods that allow the identification of their chemical forms. We recently proposed the use of Raman spectroscopy as a simple, noninvasive technique that can be used to identify and quantify melanins in feathers and hairs. Contrarily, other authors later stated that melanins are characterized by a lack of defined Raman signals. Here, we use confocal Raman microscopy to confirm previous analyses showing that the two main chemical forms of melanins (eumelanin and pheomelanin) exhibit distinct Raman signal and compare different excitation wavelengths to analyze synthetic pheomelanin and natural melanins in feathers of different species of birds. Our analyses indicate that only laser excitation wavelengths below 1064 nm are useful for the analysis of melanins by Raman spectroscopy, and only 780-nm laser in the case of melanins in feathers. These findings show that the capacity of Raman spectroscopy to distinguish different chemical forms of melanins depends on laser power and integration time. As a consequence, Raman spectroscopy should be applied after preliminar analyses using a range of these parameters, especially in fragile biological tissues such as feathers.

## Introduction

Melanins are the most prevalent biological pigments. They have primarily evolved to protect cells from the damaging effects of ultraviolet (UV) radiation, but their optical properties make that they fulfill secondary functions related to visual communication. Thus, melanins form the basis of colored traits that often evolve as intraspecific signals of genotypic quality, an issue that has been investigated mainly in birds (McGraw 2008; Guindre-Parker and Love 2014; Galván et al. 2015). Identifying the two main chemical forms of melanins (eumelanins, polymers of indole units, and pheomelanins, oligomers of sulfur-containing heterocycles; Ito et al. 2011) and quantifying them in the integument where they fulfill the signaling roles is therefore essential to understand the function and evolution of melanin-based traits.

However, the almost complete insolubility in all solvents of eumelanin and the lack of simple methods to separate eumelanins and pheomelanins make the noninvasive analysis of melanins a difficult task. Recently, however, it has been shown that Raman spectroscopy represents an effective technique to identify and quantify melanins without destroying the biological tissues where they are embedded (Galván et al. 2013a). Raman spectroscopy consists in the detection of the inelastic scattering produced when a molecule is excited with light from a ground state to a virtual energy state, so that the change in frequency of the molecule between its initial state and the state after excitation (Stokes shift) is specific to the nature of the bonds or the structure of the molecule (Colthub et al. 1990). Raman spectroscopy is used to identify a great diversity of compounds through the different features of the spectra generated by their Raman radiation and is particularly useful to identify complex samples of biological origin (e.g., Czamara et al. 2015). Additionally, the noninvasive nature of this technique makes it one of the few or even the only method applicable to the identification of very delicate systems such as rock paintings (Olivares et al. 2013). In the case of melanins, the fact that these biological pigments exhibit Raman spectra with different features (see Methods for a detailed description of Raman bands characteristic of each melanin type) allows both their identification by examining the spectral features that are present and their quantification by measuring the shape of these features (Galván et al. 2013a).

Almost concurrently, Thomas et al. (2013) stated that, when analyzed by Raman spectroscopy, melanins in feathers do not exhibit characteristic Raman spectra but spectral artifacts caused by fluorescence instead. In another study, however, some of the same authors have stated that melanins, without distinguishing different chemical forms of these pigments, exhibited a distinctive spectrum, while at the same time recognizing that it was not unequivocal evidence of the presence of melanins (Thomas et al. 2014). Therefore, confusion exists about the application of Raman spectroscopy to the analysis of melanins in the animal integument.

Thomas et al. (2013) used dispersive and Fourier transform (FT) Raman spectrometry to analyze barbs and barbules of feathers of several species of birds in an attempt to characterize different pigments (melanins, carotenoids, porphyrins, psittacofulvins, iron oxide, and a new pigment from penguins that they termed spheniscin) that appear in the avian plumage. In the case of feathers containing melanins, they found that eumelanin and pheomelanin fluoresced at both 780- and 1064-nm excitation, the former inducing an spectral artifact consisting of a sinusoidal disturbance between 200 and 1600 cm^−1^. They therefore concluded that this fluorescence response is diagnostic for melanins (Thomas et al. 2013). In another study, feathers excited with a 1064-nm laser resulted in a Raman band around 1100 nm that was assigned to melanin (Thomas et al. 2014). However, these studies generally overlooked that the Raman spectra of eumelanin had been described almost a decade ago and it included defined Raman bands at 1380 and 1580 cm^−1^ resembling the D and G bands characteristic of disordered graphite and absence of signal in the 1750–2500 cm^−1^ region (Huang et al. 2004). This Raman spectrum can be detected from eumelanin in feathers (Galván et al. 2013a). Additionally, results in Thomas et al. (2013) conflict with the ones reported by Galván et al. (2013b), who characterized the Raman spectrum of pheomelanin, again not including fluorescence but wide Raman bands at about 500, 1490, and 2000 cm^−1^ (Galván et al. 2013b). This Raman spectrum can also be detected from pheomelanin embedded in feathers (Galván et al. 2013a). Therefore, Thomas et al.'s (2013) main conclusion may not be right, as fluorescence disturbances are not the characteristic features of melanins when analyzed by Raman spectroscopy, but defined Raman bands can be found in both eumelanin and pheomelanin, including when these pigments are analyzed in feathers. Additionally, the Raman band around 1100 nm assigned by Thomas et al. (2014) to melanin does not seem to correspond to any of the Raman bands previously empirically shown to derive from the presence of melanins.

As this existing confusion may preclude the application of Raman spectroscopy by future users of the technique in either feathers or other biological tissues due to the given apparent impossibility of obtaining Raman signal from melanins, or a wrong application of the technique that is based on the search for diagnostic features consisting of fluorescence disturbances, we analyzed feather samples from the same bird species as in Thomas et al.'s (2013) study to establish which parameters of Raman spectroscopy allow the identification of melanins.

## Methods

We used laser excitation wavelengths at 780 and 1064 nm to replicate the analyses made by Thomas et al. (2013), but also at 532 nm to investigate potential effects of the excitation wavelength on the capacity to obtain Raman signal from melanins. We used feathers from the 10 following species of birds, all included in Thomas et al.'s (2013) study: red-winged blackbird *Agelaius phoenicus*, Alpine swift *Apus melba*, common raven *Corvus corax*, chaffinch *Fringilla coelebs*, common snipe *Gallinago gallinago*, nightingale *Luscinia megarhynchos*, whimbrel *Numenius phaeopus*, great cormorant *Phalacrocorax carbo*, blackcap *Sylvia atricapilla,* and common blackbird *Turdus merula*. This represents almost half (48%) the number of species considered by Thomas et al. (2013) for the analysis of melanins by Raman spectroscopy (*n* = 21). We used the indications for feather coloration and expected pigment (i.e., eumelanin or pheomelanin) given in the supplementary material of Thomas et al. (2013) to determine the particular plumage patches from which feather samples had to be taken. All feather samples were collected from museum skins deposited in the bird collection of the Museo Nacional de Ciencias Naturales (MNCN-CSIC, Madrid, Spain). We analyzed one feather from one specimen for each species because the characteristics of the Raman spectra of melanins are highly repeatable between different feathers of the same birds (Galván et al. 2013a), focusing the laser beam at one barb and one barbule of each feather. This method has been proved efficient at distinguishing eumelanin and pheomelanin when both melanin forms are mixed in the focal tissues, as it usually happens (Galván et al. 2013a,b). We also analyzed a barb of an unpigmented feather of a white Gyrfalcon *Falco rusticolus* as a control, as the Raman signal from keratin (the main compound in nonpigmented feathers; Hsu et al. 1976) should differ from that of melanins.

We used a Thermo Fisher DXR confocal dispersive Raman microscope (Thermo Fisher Scientific, Madison, WI, USA) operating in MNCN-CSIC (Madrid, Spain) with a point-and-shoot Raman capability of 1-*μ*m spatial resolution and using excitation laser sources at 532 nm of 1 mW power and at 780 nm of 4 mW power. The single spectra were obtained using a 100× MPlan achromat confocal objective lens (Olympus, Hamburg, Germany) confocal objective, a slit aperture of 50 *μ*m, and a grating of 400 lines/mm. These conditions produced an average spectral resolution of 2.2–4.4 cm^−1^ in the wavenumber range of 100–2500 cm^−1^. An integration time of 3 sec × 16 accumulations allowed getting an acceptable SNR and a photobleaching time of 10 sec. Laser power and integration time values were chosen to optimize SNR values in each type of sample while avoiding damage to them (breakage of the focal feather barbs and barbules), which the confocal microscope allowed us to determine in real time. The system was operated with Thermo Fisher OMNIC 8.1 software. The dispersive Raman spectra reported by Thomas et al. (2013) were obtained using an excitation wavelength of 780 nm with a Nicolet Almega XR spectrometer (Thermo Electron Corporation, Madison, WI) equipped with a 150 mW diode laser and a Peltier-cooled CCD detector, and spectra were collected through a 50× or 100× Mplan apochromatic objective lens (Olympus, Melville, NY) and 50- or 100-mm pinhole aperture in a BX51 confocal microscope (Olympus). Each spectrum was a co-addition of 32 scans across 200–3400 cm^−1^ (2.6–4.9 cm^−1^ resolution). Magnification, aperture, and laser power were optimized between analyses to minimize spectral noise and to maximize spectral signal from samples with a range of energy tolerances. Their system was operated with Thermo Fisher OMNIC 8.2 software. Both Thomas et al. (2013) and us focused the laser beam to barbs and barbules of the feathers. Therefore, both our equipment and the processing of the resulting spectra were very similar to those of Thomas et al.'s (2013) study.

In addition to the dispersive Raman spectrometer, we used a FT-Raman spectrometer to analyze pheomelanin from feathers (in this case, orange flank feathers from a zebra finch *Taenyopigia guttata*, as degradative analyses have shown that the color of these feathers is due to the presence of pheomelanin; McGraw and Wakamatsu 2004) and compare the results with those obtained with dispersive Raman. We also analyzed synthetic pheomelanin derived from 5-S-cysteinyldopa (5SCD), the most abundant cysteinyldopa isomer that is formed when cysteine is added to dopaquinone (i.e., the first step of the pheomelanogenesis pathway), synthesized from DOPA (3, 4-dihydroxyphenylalanine) and cysteine using tyrosinase as a natural oxidant following Wakamatsu et al. (2009). FT-Raman spectra were obtained using a Perkin-Elmer System 2000R Fourier transform Raman system coupled to a System GX-2000 interferometer, using a Nd^3+^:YAG laser source with a 1064 nm exciting line and a room temperature InGaAs detector. The spectra were recorded with a resolution of 4 cm^−1^ accumulating 200 scans (OPD velocity 0.1 cm sec^−1^) and corrected for the optical characteristics of the instrument using a low energy tungsten lamp. Relatively low laser powers of between <15 mW were necessary to reduce absorption of the laser and to maintain the high luminescence background within the dynamic range of the detector. Thomas et al. (2013) also obtained FT-Raman spectra of feathers, which were collected with a Nicolet Almega XR spectrometer and using an NXR FT-Raman module coupled to a 6700 FTIR spectrometer (Thermo Electron Corporation). The Raman module was equipped with a Nd:YVO_4_ laser, a CaF_2_ beam splitter, and a Peltier-cooled InGaAs detector, and the spectra were collected using the 1 mm laser spot of a microstage, each spectrum being a co-addition of 1024 scans across 100–3700 cm^−1^ (4 cm^−1^ resolution), with laser power set at 0.2, 1 or 1.4 W depending on the energy tolerance of the feather barbs (Thomas et al. 2013).

## Results and Discussion

**Figure 1:**
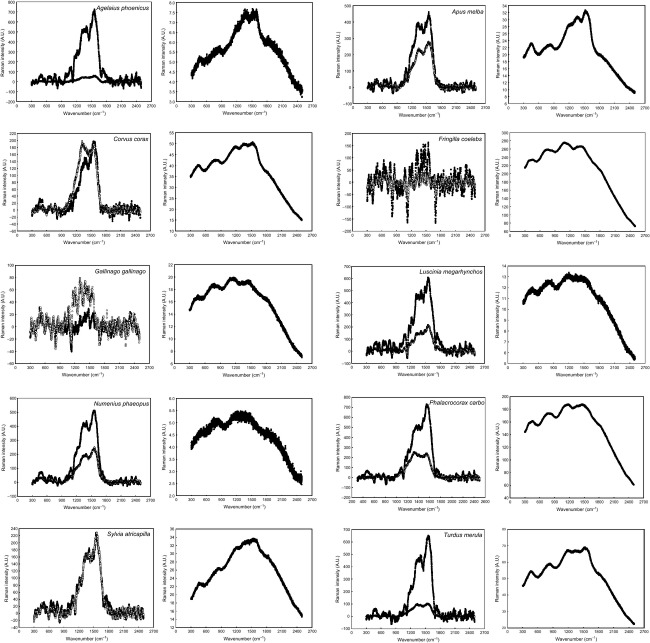
Spectra of melanins in the feathers of 10 species of birds obtained by dispersive Raman spectroscopy with laser excitation at 780 nm. In each pair of graphs, the left one corresponds to this study and the right one corresponds to the study by Thomas et al. (2013). All spectra correspond to eumelanin excepting those of *F. coelebs* and *G. gallinago* that correspond to pheomelanin. Raw spectral data without baseline correction nor smoothing are shown. Solid symbols: barbs, open symbols: barbules.

**Figure 2:**
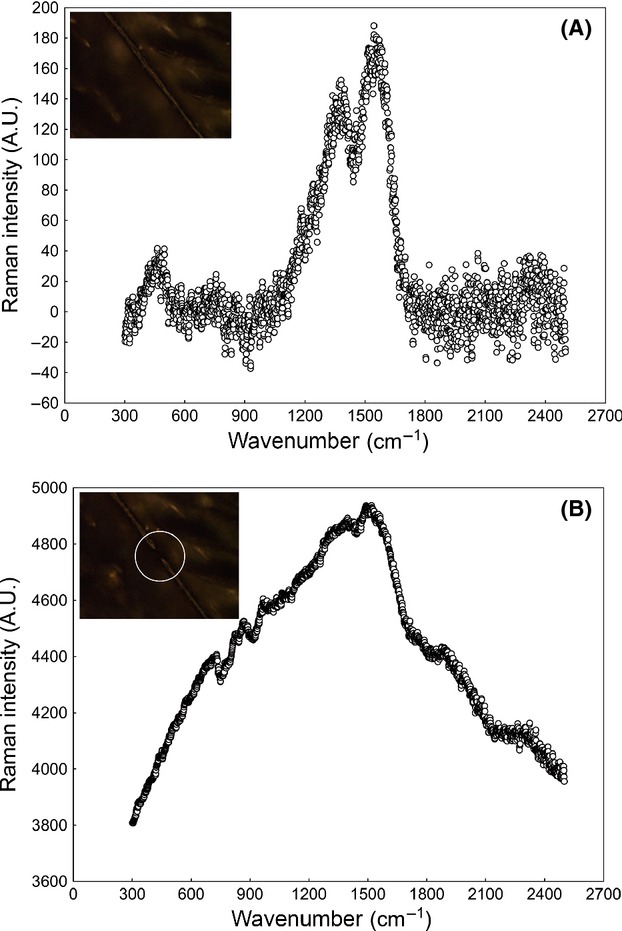
(A) Raman spectrum of eumelanin obtained from a barb using a laser power of 2 mW. (B) Fluorescence disturbance obtained when the same barb was broken due to an excess laser power (10 mW). Inserts are confocal microscope images showing the barb before (A) and after (B) breakage. The white circle in B indicates the point where the barb was broken. The barb corresponds to a dark gray feather from the back of a Northern goshawk *Accipiter gentilis*. Raw spectral data without baseline correction nor smoothing are shown.

When we analyzed the feathers with dispersive Raman and excitation wavelength at 532 nm, the laser beam broke all focused feather barbs and barbules even at the lowest intensity possible (0.1 mW, the lowest setting on the instrument), and no Raman spectra could be obtained. Raman spectra of melanins could only be obtained when the excitation wavelength was set at 780 nm. Figure1 shows the dispersive Raman spectra with excitation wavelength at 780 nm that we obtained in comparison with those obtained by Thomas et al. (2013) for the same species of birds and the same type of feathers. We obtained the previously reported characteristic Raman spectra for eumelanin and pheomelanin (Galván et al. 2013a,b) in the species in which Thomas et al. (2013) expected to find either eumelanin or pheomelanin, respectively, based on the color of feathers. Our spectra were very different from those obtained by Thomas et al. (2013), as while we found the Raman bands at about 1380 and 1580 cm^−1^ and absence of signal in the 1750–2500 cm^−1^ region characteristic of eumelanin (Huang et al. 2004; also a band about 500 cm^−1^ described by Galván et al. (2013a)), and the wide Raman bands at about 500, 1490, and 2000 cm^−1^ characteristic of pheomelanin (Galván et al. 2013b), the spectra reported by Thomas et al. (2013) all exhibited a sinusoidal disturbance characteristic of fluorescence in the range 300–1600 cm^−1^ irrespective of the melanin type present in the feather (i.e., no difference was reported between eumelanin and pheomelanin) (Fig.1). The characteristic Raman bands of eumelanin in our spectra were, however, glimpsed in some of Thomas et al.'s spectra (those corresponding to *A. phoenicus* and *A. melba*, Fig.1). Thus, our findings contradict the study of Thomas et al. (2013) and show that the Raman spectra of both eumelanin and pheomelanin can be detected in feathers when analyzed with excitation wavelength at 780 nm. Our dispersive Raman equipment was very similar to that of Thomas et al. (2013), but it is noticeable that they set a laser power of 150 mW and 32 accumulations while both our laser power (4 mW) and integration time (16 accumulations) that were set were significantly lower. It is then possible that Thomas et al. (2013) damaged the feather barbs and barbules using too large laser power and integration time without noticing it and this is the cause of the spectral artifacts that they found. Indeed, we broke several barbs and barbules in our samples before reaching the optimal values of 4 mW laser power and 16 accumulations, and we found the sinusoidal fluorescence disturbances reported by Thomas et al. (2013) every time the samples were broken. Figure2 shows how the typical Raman signal from melanin disappears due to the fluorescence disturbances that are generated when the laser power is increased in excess and the feather is broken.

**Figure 3:**
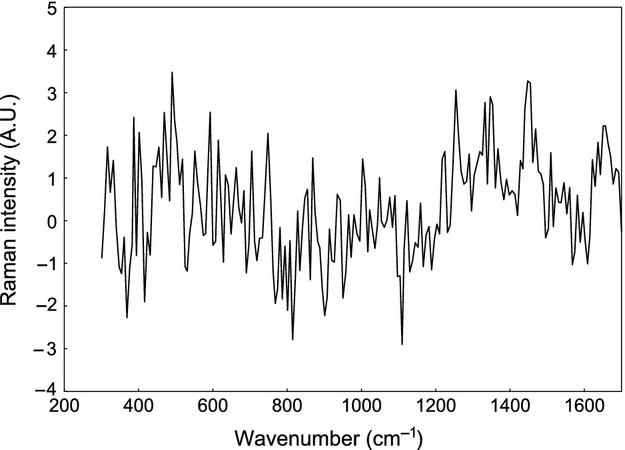
Raman spectrum of keratin from a barb of an unpigmented feather of a Gyrfalcon. Given the high number of bands generated by keratin and the relatively complex Raman spectrum that results (Hsu et al. 1976), a curve fitted to the spectral points is shown instead of the raw data to facilitate visualization of the spectrum. The function was fitted using the bicubic spline smoothing procedure.

Lastly, an unpigmented feather of a Gyrfalcon showed a Raman spectrum completely different to the spectra of melanins and also to those obtained when the samples were broken (Fig.3). Indeed, the latter was characteristic of keratin, the protein where melanins are embedded in feathers (Hsu et al. 1976). Thus, none of our results can be attributed to neither fluorescence disturbances nor to keratin Raman signal, but to melanins, which show distinctive Raman bands.

**Figure 4:**
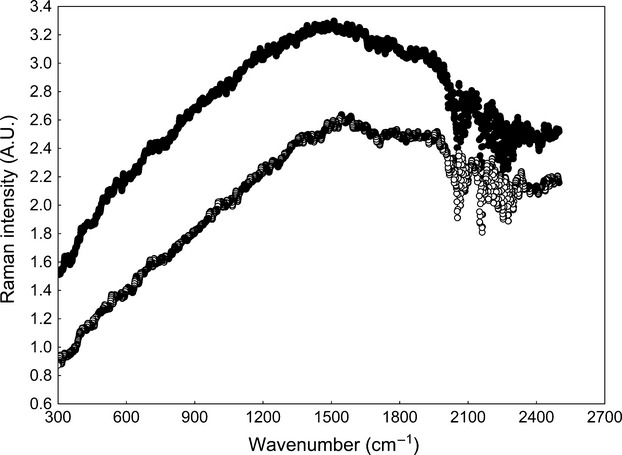
Spectra of synthetic pheomelanin (solid symbols) and natural pheomelanin in a flank feather of a male zebra finch (open symbols) obtained by FT-Raman spectroscopy with laser excitation at 1064 nm. Raw spectral data without baseline correction nor smoothing are shown.

On the other hand, we could not detect Raman signal of pheomelanin when we analyzed by FT-Raman and wavelength excitation at 1064 nm the synthetic pheomelanin or the pheomelanin-containing flank feather of a male zebra finch, and low-intensity Raman signal with no defined bands was found (Fig.4). This is very similar to the spectra of melanins obtained by FT-Raman and wavelength excitation at 1064 nm that were reported by Thomas et al. (2013) in the feathers of several species of birds. We did not analyze eumelanin by FT-Raman, but as there are two main groups of melanins (eumelanin and pheomelanin), the fact that at least one of them cannot be detected by FT-Raman is enough to make 1064-nm laser useless for the analysis of melanins. Both the synthetic pheomelanin and the natural pheomelanin from zebra finch feathers exhibit characteristic Raman signal when analyzed by dispersive Raman and wavelength excitation at 780 nm (Galván et al. 2013b). It also confirms that the Raman band around 1100 nm found by Thomas et al. (2014) in feathers embedded in amber after excitation with a 1064-nm laser is equivocal evidence of melanin presence, as the authors suggested.

Our findings indicate that the Raman signal of melanins in feathers can only be obtained with laser wavelength excitation at 780 nm, and neither lower (532 nm) nor higher (1064 nm) excitation wavelengths are useful for the analysis of feather melanins by Raman spectroscopy. Excitation wavelengths differ in their capacity to detect Raman bands, which greatly depends on the structural nature of the analyzed compounds (e.g., Wood and McNaughton 2002). In our case, however, the incapacity of 532 nm wavelength to obtain Raman spectra of melanins in feathers was due the fragility of the structures where melanins are embedded (i.e., feather barbs and barbules), which are broken by the high energy of the laser beam at such wavelength. Huang et al. (2004) were able to obtain Raman signal of synthetic eumelanin and natural eumelanin from *Sepia* with excitation wavelengths even below 532 nm (457.9 and 514.5 nm), which confirms that our limitation to obtain Raman spectra of melanins with excitation wavelength at 532 nm was due to the fragility of feathers and not to structural properties of melanins. By contrast, the incapacity of 1064-nm excitation wavelength to obtain Raman spectra of melanins shown here, confirming previous findings by Thomas et al. (2013), was independent of the nature (natural or synthetic) of the pigments, which suggests that only lower excitation wavelengths (i.e., those normally used in dispersive Raman) are useful for the analysis of melanins by Raman spectroscopy. This is probably because the energy of 1064-nm lasers is too low to generate a detectable number of inelastically scattered photons from melanin polymers.

In conclusion, our analyses indicate that only the laser excitation wavelengths that are normally used by dispersive Raman (<1064 nm) seem to be useful for the analysis of melanins by Raman spectroscopy, and only 780-nm laser in the case of melanins in feathers. Adjusting laser power and integration time appears as crucial for obtaining Raman bands characteristic of eumelanin and pheomelanin and avoiding spectral artifacts caused by fluorescence, especially in fragile biological tissues such as feathers. Given the consistency in the shape of Raman spectra of melanins in both natural and synthetic pigments, in melanins from vertebrates and invertebrates, and in different tissues (Huang et al. 2004; Galván et al. 2013a,b), Raman spectroscopy has the potential to become a universal tool for the analysis of the so far elusive melanins.
